# Potential Anticancer Activity of the Furanocoumarin Derivative Xanthotoxin Isolated from *Ammi majus* L. Fruits: In Vitro and In Silico Studies

**DOI:** 10.3390/molecules27030943

**Published:** 2022-01-29

**Authors:** Marwa Y. Issa, Mohamed F. Elshal, Noha Fathallah, Mostafa A. Abdelkawy, Mokhtar Bishr, Osama Salama, Yasmin S. Abulfadl

**Affiliations:** 1 Pharmacognosy Department, Faculty of Pharmacy, Cairo University, Cairo 11562, Egypt; mostafa.abdelkawy@pharma.cu.edu.eg; 2 Molecular Biology Department, Genetic Engineering and Biotechnology Institute, University of Sadat City, Sadat City 32897, Egypt; mohamed.elshal@gebri.usc.edu.eg; 3 Medicinal Plants Department, Faculty of Pharmacy, Future University in Egypt, Cairo 11835, Egypt; noha.mostafa@fue.edu.eg (N.F.); osalama99@hotmail.com (O.S.); 4 Arab Company for Pharmaceuticals and Medicinal Plants, El-Sharkya 11361, Egypt; mbishr_2000@yahoo.com; 5 Department of Pharmacology, Toxicology, and Biochemistry, Faculty of Pharmacy, Future University in Egypt, Cairo 11835, Egypt; yasmine.saad@fue.edu.eg

**Keywords:** *Ammi majus*, coumarins, xanthotoxin, apoptosis, HepG2, molecular docking, topoisomerase II

## Abstract

*Ammi majus* L., an indigenous plant in Egypt, is widely used in traditional medicine due to its various pharmacological properties. We aimed to evaluate the anticancer properties of *Ammi majus* fruit methanol extract (AME) against liver cancer and to elucidate the active compound(s) and their mechanisms of action. Three fractions from AME (Hexane, CH_2_Cl_2_, and EtOAc) were tested for their anticancer activities against HepG2 cell line in vitro (cytotoxicity assay, cell cycle analysis, annexin V-FITC apoptosis assay, and autophagy efflux assay) and in silico (molecular docking). Among the AME fractions, CH_2_Cl_2_ fraction revealed the most potent cytotoxic activity. The structures of compounds isolated from the CH_2_Cl_2_ fraction were elucidated using ^1^H- and ^13^C-NMR and found that Compound **1** (xanthotoxin) has the strongest cytotoxic activity against HepG2 cells (IC_50_ 6.9 ± 1.07 µg/mL). Treating HepG2 cells with 6.9 µg/mL of xanthotoxin induced significant changes in the DNA-cell cycle (increases in apoptotic pre-G1 and G2/M phases and a decrease in the S-phase). Xanthotoxin induced significant increase in Annexin-V-positive HepG2 cells both at the early and late stages of apoptosis, as well as a significant decrease in autophagic flux in cancer compared with control cells. In silico analysis of xanthotoxin against the DNA-relaxing enzyme topoisomease II (PDB code: 3QX3) revealed strong interaction with the key amino acid Asp479 in a similar fashion to that of the co-crystallized inhibitor (etoposide), implying that xanthotoxin has a potential of a broad-spectrum anticancer activity. Our results indicate that xanthotoxin exhibits anticancer effects with good biocompatibility toward normal human cells. Further studies are needed to optimize its antitumor efficacy, toxicity, solubility, and pharmacokinetics.

## 1. Introduction

Hepatocellular carcinoma (HCC) is the sixth and the fourth common cancer in the world and in Egypt, respectively [1]. HCC represents one of the most aggressive solid tumors and the third leading cause of cancer-related mortality worldwide. The prognosis of patients with HCC is poor, with around 80% mortality rate within one year from the first diagnosis and an overall 5-years survival rate of 18% [2].

Despite decades of basic and clinical research and trials of systemic chemotherapy for HCC including cisplatin, doxorubicin, and 5-flurouracil, which are used as mono- or combined therapy, only 10 to 25% response rate with marginal survival improvement have been reached mainly due to development of resistance and recurrence [3]. On the other hand, several medicinal plants and dietary phytochemicals have emerged as an initiative therapeutic option for HCC management because of their abilities in targeting multiple molecular targets in disease signaling pathways [4]. In fact, most of the current chemotherapeutic drugs are from natural products origin such as doxorubicin, paclitaxel, vincristine, etc. [5]. Today, bioactive compounds derived from foods and plant sources play an important role in the prevention and treatment protocols of HCC [6]. Among the other advantages of natural drugs are their easy availability and economic value; however, the climbing demands for these rich and potent agents is based on their efficacy to scavenge free radicals, stimulate apoptosis, and inhibit cancer cells’ survival [7].

*Ammi majus* L. (Apiaceae), growing in Egypt, is famous for its content of active constituents as coumarins, flavonoids, and steroids [8]. The fruits are primarily used, which contain furanocoumarins, widely used in skin conditions such as vitiligo and psoriasis [9]. Several bioactive compounds and various biological activities of *A. majus* have been reported including antioxidant, antibacterial, antifungal, and cytotoxic activities [10]. However, there are no studies that show the cytotoxic activity of the fruits of this plant and its isolated major furanocoumarin, namely, xanthotoxin on liver cancer cell lines, so far. Therefore, we aimed to investigate the antiproliferative/cytotoxic, cell cycle regulation, apoptosis-inducing, and autophagy interfering profile of the main active constituent of *A. majus* methanol extract against liver cancer cell line HepG2.

## 2. Results

### 2.1. Cellular Cytocompatibility and Cytotoxicity of AME and Its Fractions

**Figure 1:**
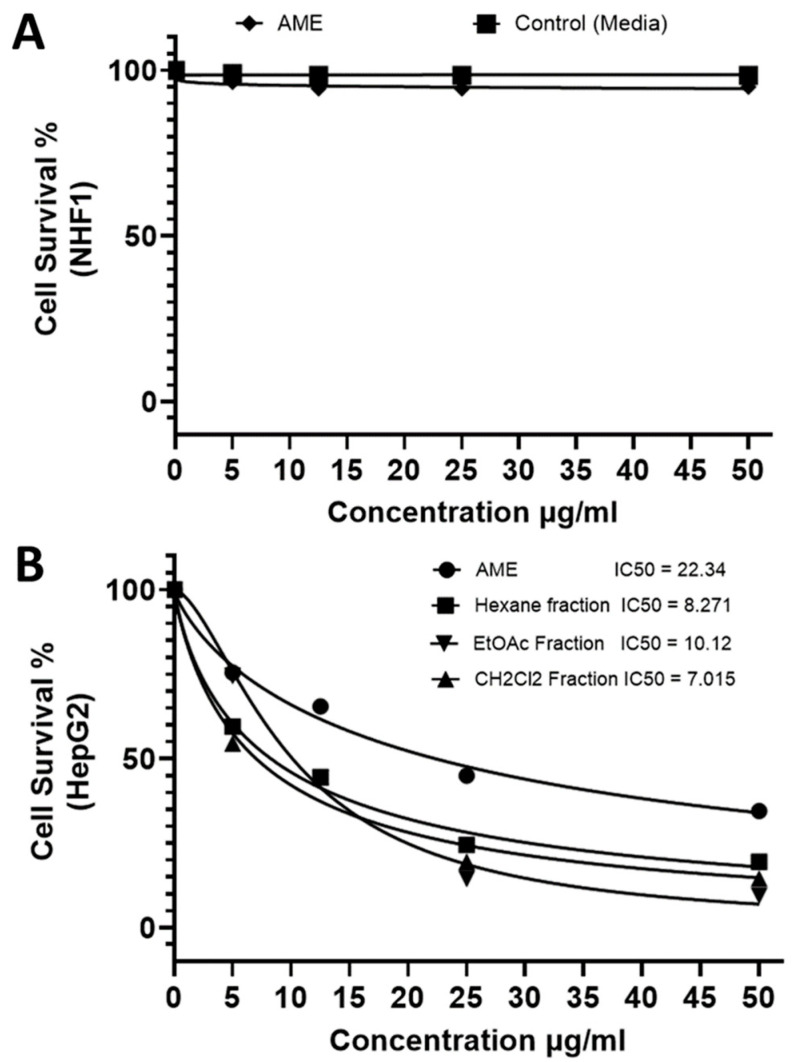
In vitro cytocompatibility and cytotoxicity assays. Sulforhodamine B (SRB) colorimetric assay for cytotoxicity screening evaluated on normal human fibroblast cells (NHF1) (**A**) and liver cancer cells (HepG2) (**B**) seeded at 2 × 10^3^ cells for 48 h in complete DMEM. All determinations were carried out in triplicates, and values are expressed as means ± SEM.

The crude methanol extract of *A. majus* fruits (AME) was tested for its cytocompatibility and cytotoxicity on normal human fibroblastic cell line (NHF1) and liver cancer cell line (HepG2), respectively. Cells cultured with DMSO plus medium alone were counted as control; results were conveyed as cell survival % relative to 100% of controls. The cytocompatibility assessment of the total extract on normal human fibroblast cells, proved that it is highly discriminating between cancer and normal cells, which means high selectivity toward the abnormal cancerous cells when compared to control. AME showed about 5% killing of normal cells with the maximum applied concentration (Figure 1A). The AME was thus claimed to be essentially nontoxic and to have a wide safety margin.

The in vitro testing of the cytotoxic potential of the AME extract and its fractions on HepG2 (human hepatocellular carcinoma cell line) was evaluated. Among the tested fractions, those with the highest potency were CH_2_Cl_2_ (IC_50_ 7.015 μg/mL) and hexane, then ethyl acetate fractions (Figure 1B).

### 2.2. Identification and Elucidation of the Isolated Compounds

**Figure 2:**
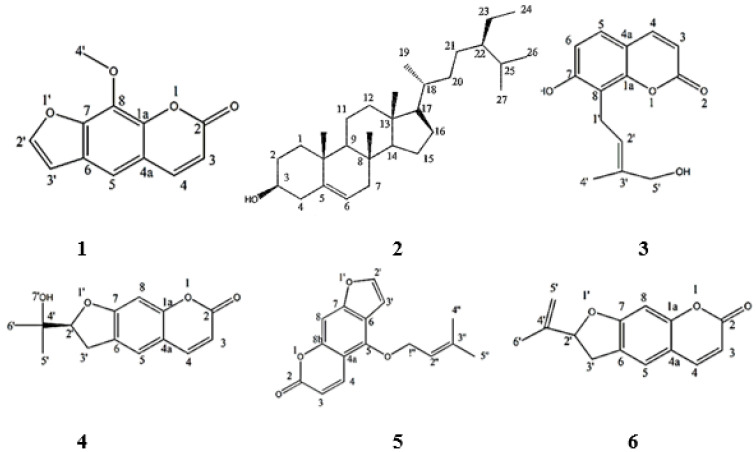
Compounds isolated from the CH_2_Cl_2_ fraction of AME. Xanthotoxin (**1**), β-sitosterol (**2**), Isoarnottinin (**3**), Marmesin (**4**), Imperatorin (**5**), Ammirin (**6**).

Dichloromethane fraction was used for isolation of its major compounds (Figure 2) having the highest cytotoxic activity. Its fractionation resulted in the production of two fractions, fraction A (200 mg), which yielded three compounds (**1** (12 mg), **2** (13 mg), and **3** (10 mg)), and fraction B (170 mg), which produced further three compounds (**4** (13 mg), **5** (17 mg), and **6** (20 mg)). The isolated compounds were identified based on their physical characteristics, spectral data analysis (^1^H- and ^13^C-NMR, Supplementary material) (Table 1a,b), and literature comparison.

- Xanthotoxin

Compound **1** was isolated from fraction A as white needle crystals, and it gave a violet spot upon spraying with vanillin sulfuric acid with R_f_ = 0.5 in CHCl_3_-CH_3_OH (95:5). It is soluble in organic solvents such as CH_2_Cl_2_, and ethanol, and it is insoluble in water. The ^1^H-NMR spectrum defined all the eight protons. The spectrum revealed a typical furanocoumarin skeleton with two doublets at protons H-3 and H-4 at (δ_H_ 6.38, d, *J* = 12 Hz, 1H) and (δ_H_, 7.78 d, *J* = 12 Hz, 1H), which are typical of a coumarin nucleus unsubstituted in the pyrone ring, and the presence of one-proton singlet at H-5 (δ_H_ 7.37, s, 1H). The signal consisting of three protons H- 4′ (δ_H_ 4.3, s, 3H) is assigned to one aromatic methoxyl group. The two doublets at (δ_H_ 7.70, d, *J* = 4 Hz, 1H) and (δ_H_ 6.83, d, *J* = 4 Hz, 1H) for H-2′ and H-3′, respectively, are typical of the unsubstituted furan ring. The ^13^C-NMR spectrum revealed the presence of carbon signals classified as six quaternary, five methine CH, and one methyl CH_3_ carbons; the spectrum showed some similarities to coumarins [11] with a methoxy substitution at (δ_C_ 132.8, C-8). More characteristic signals including the carbonyl (δ_C_ 161.8, C-2) and (δ_C_ 147.8, C-2′) were observed, and thus, after reviewing the literature, it was confirmed that it is 8-methoxy psoralen (Xanthotoxin), which is the main active constituent of *A. majus* [12].

- β–sitosterol

Compound **2** was acquired from fraction A as white amorphous powder. The ^1^H-NMR spectrum revealed a typical spectrum for sterols, as shown in Table 1b. The olefinic signal at (δ_H_ 5.37, m, 1H) appeared to be characteristic of the sterols, and it was assigned to H-6 proton in the β–sitosterol chemical nucleus. The ^1^H-NMR spectrum of this compound also exhibited a signal corresponding to the proton connected to the C-3 hydroxyl group, which appeared as a multiplet at (δ_H_ 3.55, m, 1H). More proton signals were evident of the sterol nucleus as the secondary methyl groups (δ_H_ 0.85, d, *J* = 1.8 Hz, 3H), (δ_H_ 0.88, d, *J* = 8.0 Hz, 3H), and (δ_H_ 0.94, d, *J* = 6.5 Hz, 3H) for H-27, H-26, and H-21, respectively, which are characteristic at the up field area [13]. After reviewing the literature and comparing the spectra, the compound was identified as β–sitosterol, which was isolated before from *A. majus* [14].

- Isoarnottinin

Compound **3** was obtained from fraction A as a faint yellow powder. The ^1^H-NMR spectrum of 3 revealed some signals that were similar to the signals characterizing coumarin nucleus [15] and showed partial resemblance with **1**, especially at (δ_H_ 7.62, d, *J* = 8.5, 1H, H-4) and (δ_H_ 7.05, d, *J* = 8.0 Hz, 1H, H-5), corresponding to the two unsubstituted aromatic carbons, but it lacked the signals of the furan ring at H-2′ and H-3′. However, the prenyl group attached to C-8 of the benzene ring gave some characteristic signals at (δ_H_ 5.37, m, 1H, H-2′), (δ_H_ 1.7, s, 3H, H-4′), and (δ_H_ 4.20, s, 2H (OH), H-5′). The spectra contained five well-established doublets at (δ_H_ 3.57, 6.08, 7.62, 7.05, and 7.27 at H-1′, H-3, H-4, H-5, and H-6), respectively [16]. After reviewing the literature, it was suggested as Isoarnottinin, previously isolated from *A. majus* [17].

- Marmesin

Compound **4** was isolated from fraction B as a buff amorphous solid. ^1^H-NMR spectrum exhibited some similarities with **1** that confirmed the presence of a furanocoumarin nucleus, with some differences at the furan ring that revealed saturation at (δ_H_ 4.21, m, 1H, H-2′) and (δ_H_ 2.42, d, *J* = 0.8 Hz, 2H, H-3′), respectively. Another difference was observed with the hydroxypropyl side chain giving signals at (δ_H_ 1.28, s, 3H, H-5′) and (δ_H_ 1.65, s, 3H, H-6′). The ^13^C-NMR spectrum revealed the presence of 14 carbon signals including 7 C, 4 CH, 1 CH_2_, and 2 CH_3_; the spectrum showed similarities to Compound **1** with some differences at the hydroxypropyl side chain, where it showed aliphatic CH_3_ groups on C-5′ to C-6′. The downfield signal at (δ_C_ 169.9, C-2) is very characteristic of the carbonyl group. The signals at (δ_C_ 95.9 and 21.5) of C-2′ and C-3′, respectively, indicated the absence of the double bond in the furan ring. Compound **4** was identified as Marmesin, formerly isolated from *A. majus* fruits [18], and was proved to have important biological activities [19].

- Imperatorin

Compound **5** was obtained from fraction B as off-white, long needles with a melting point of 100 °C. ^1^H-NMR spectrum exhibited some resemblances with **1** that confirmed the presence of a similar furanocoumarin nucleus with some differences at carbon number 5 with a substitution signal at (δ_H_ 4.33, d, *J* = 2.3 Hz, 2H, H-1″), (δ_H_ 5.39, m, 1H, H-2″), (δ_H_ 1.28, s, 3H, H-4″), and (δ_H_ 1.57 s, 3H, H-5″), respectively. Another difference was observed at (δ_H_ 7.38, s, 1H, H-8). The two doublets at (δ_H_ 6.40, d, *J* = 9.5 Hz, 1H) and (δ_H_ 7.79, d, *J* = 9.6 Hz, 1H) for H3 and H4, respectively, are characteristic of the ortho coupling with the carbonyl group and confirmed the coumarin nucleus, while the two doublets at (δ_H_ 7.72, d, *J* = 2.3 Hz, 1H) and (δ_H_ 6.84, d, *J* = 2.2 Hz, 1H) for H-2′ and H-3′, respectively, indicated the presence of the furan ring. After investigating the spectrum and reviewing the literature, Compound **5** was identified as Imperatorin, previously isolated from *A. majus* fruits and was demonstrated to have important biological activities [20].

- Ammirin

Compound **6** was isolated from the dichloromethane extract fraction B. The ^1^H-NMR spectrum revealed evidence of α-pyrone protons on C-3 and C-4 (δ_H_ 7.05, d, *J* = 7.2 Hz, 1H and δ_H_ 7.61 d, *J* = 7.2 Hz, 1H), demonstrating the presence of a dihydrofuran system, which has an isopropenyl side chain on the C-2′; it is fused linearly onto the coumarin part. This was indicated by the two sharp aromatic proton singlets (δ_H_ 7.28 and 7.28) of carbon C-5 and C-8, respectively. The isopropyl side chain attached to C-2′ signals were observed at (δ_H_ 6.07, s, 2H) and (δ_H_, 1.27, s, 3H) for H-5′ and H-6′, respectively. The ^13^C-NMR exhibited a pattern with similarities to **4** with some differences at the isopropenyl sidechain, giving an olefinic carbon at (δ_C_, C-5′ 115.47, CH_2_) and an aliphatic carbon signal at (δ_C_, C-6′, 20.10, CH_3_). After reviewing the literature and comparing the spectrum, the compound was identified as Ammirin, previously isolated from *A. majus* fruits [21].

### 2.3. Cytotoxic Activity of Xanthotoxin against HepG2 Cell Line

**Figure 3:**
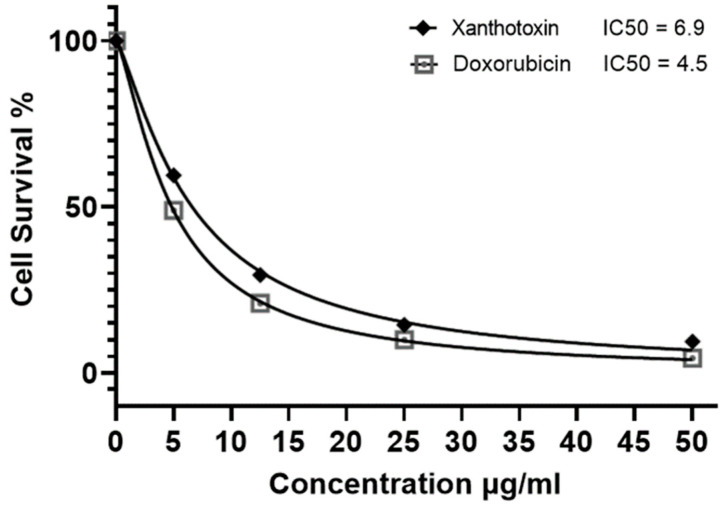
IC_50_ μg/mL of the major compound xanthotoxin isolated from *Ammi majus* fruit methanol extract against HepG2 cells, seeded at 2 × 10^3^ cells for 48 h in complete DMEM.

After purification and isolation of the compounds from fraction CH_2_Cl_2_, the major compound namely xanthotoxin was assessed against the HepG2 cell line with the same procedures, and it was observed that it exhibited potent cytotoxic activity when compared to the rest of the isolated compounds, with an IC_50_ of 6.9 μg/mL approaching that of the standard drug doxorubicin (IC_50_ 4.58 ± 0.9 μg/mL), as shown in Figure 3.

### 2.4. The Effects of Xanthotoxin on Cell Cycle Kinetics

**Figure 4:**
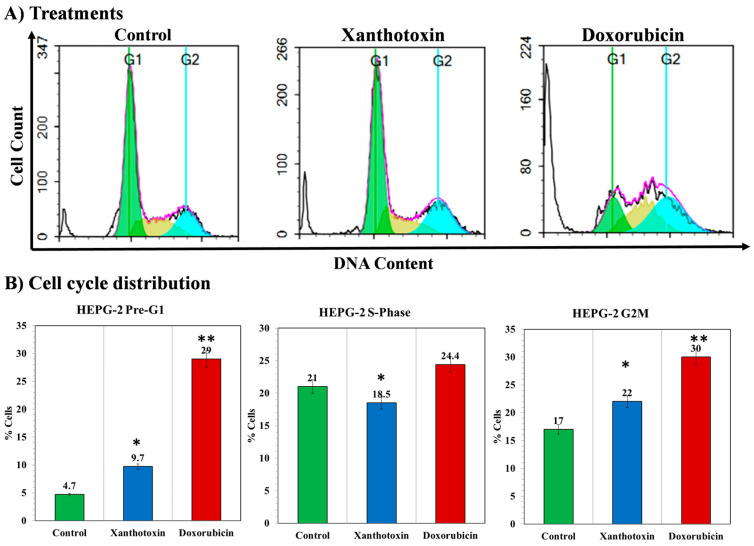
A graphical representation of cell cycle analysis of the HepG2 cells seeded at 2 × 10^3^ cells for 48 h in complete DMEM after different treatments. (**A**) Flow cytometric histograms representing the cell cycle phases after treatment with xanthotoxin against HepG2 and doxorubicin at their IC_50_ values compared with control cells. (**B**) Diagrams of the percentage of pre-G1 phase, S-phase, and G2/M phases for xanthotoxin and doxorubicin in comparison with control cells. All determinations were carried out in triplicates, and values are expressed as means ± SEM. *, ** denote significant difference at *p* < 0.05 and *p* < 0.01, respectively.

The cell cycle kinetics was analyzed to determine the mechanism of cytotoxic activity of the most active molecule against HepG2 cells by using DNA flow cytometric analysis. Xanthotoxin was studied on HepG2 cells, as it was found the most cytotoxic compound. Treatment of HepG2 cells with IC_50_ dosage of xanthotoxin induced significant alterations in cell cycle profile including a significant increase in the percentage of cell population at the G2/M phase from (17.12%) control to (22.31%). In addition, treatment of HepG2 with xanthotoxin induced significant decrease in the DNA synthesis phase (18.5%) compared to (21%) in control cells. Moreover, treatment with xanthotoxin caused a significant increase in the percentage of cells at pre-G1 phase from (4.7%) (control) to (9.7%). Therefore, it can be concluded that xanthotoxin inhibited the cell proliferation of HepG2 cells via cell cycle arrest at the G2/M phase and induction of apoptosis (Figure 4).

### 2.5. The Effects of Xanthotoxin on Programmed Cell Death

**Figure 5:**
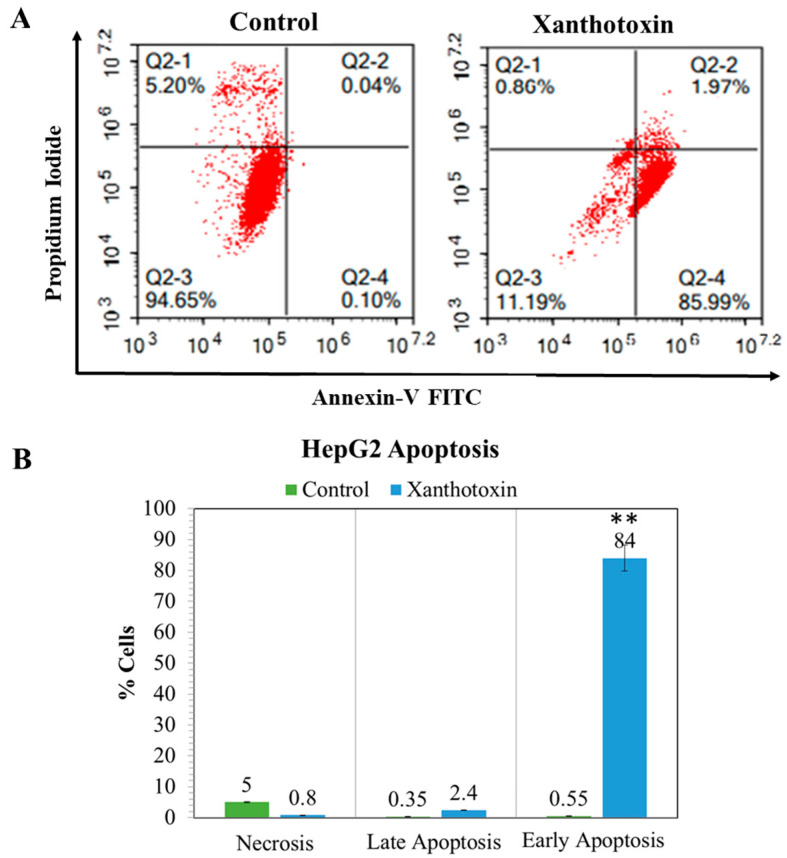
A graphical representation of the programmed cell death of the HepG2 cells after treatment with xanthotoxin. (**A**) Flow cytometric histograms representing the annexin-V/PI staining of HepG2 seeded at 2 × 10^3^ cells for 48 h in complete DMEM and treated with 6.9 µg/mL of xanthotoxin compared with control cells. (**B**) Diagrams of the percentage of necrosis and apoptosis for xanthotoxin in comparison with control cells. All analyses were carried out in triplicates, and values are expressed as means ± SEM. ** denotes significant difference at *p* < 0.01.

The effect of xanthotoxin on the induction of apoptosis in HepG2 cells was also studied using annexin-v/PI flow cytometry assay. Treatment of HepG2 cells with 6.9 µg/mL of xanthotoxin induced significant increase in the percentage of cell population at the early and late apoptosis phases from (84.32 ± 2.7 and 2.4 ± 0.13%) compared with control (0.31 ± 0.03 and 0.53 ± 0.12%), respectively. Therefore, it can be concluded that xanthotoxin inhibited the cell proliferation of HepG2 cells via cell cycle arrest at the G2/M phase and induction of apoptosis (Figure 5).

### 2.6. The Effects of Xanthotoxin on Cellular Autophagy

**Figure 6:**
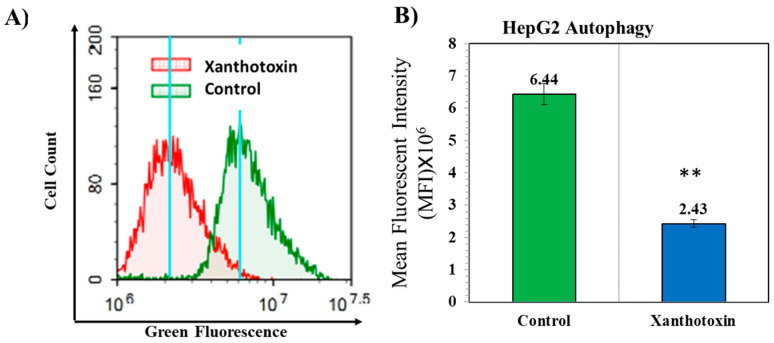
A graphical representation of autophagy in HepG2 cells treated with xanthotoxin. (**A**) Flow cytometric histograms representing the acridine orange autophagy flux assay of HepG2 seeded at 2 × 10^3^ cells for 48 h in complete DMEM and treated with 6.9 µg/mL xanthotoxin compared with control cells. (**B**) Diagrams of the percentage of necrosis and apoptosis for xanthotoxin in comparison with control cells. All analyses were carried out in triplicates, and values are expressed as means ± SEM. ** denote significant difference at *p* < 0.01.

It has been reported recently that autophagy induces pro-survival signals that confer protection to cancer. Molecules inhibiting autophagy are important for effective targeting of cancer cells [22]. To assess the effect of xanthotoxin on autophagy, HepG2 cells were cultured with 6.9 µg/mL concentration of xanthotoxin for 48 h, and acridine orange flow cytometry flux was measured to determine autophagy. We found a significant decrease in autophagic flux (fusion of autophagosomes and autolysosomes) as indicated by lower acridine orange fluorescence (MFI = 2.43 × 10^6^) compared with that of control cells (MFI = 6.44 × 10^6^) (Figure 6). These results suggest that xanthotoxin negatively affected autophagy.

### 2.7. Molecular Docking of Xanthotoxin on Topoisomerase IIb Enzyme

**Figure 7:**
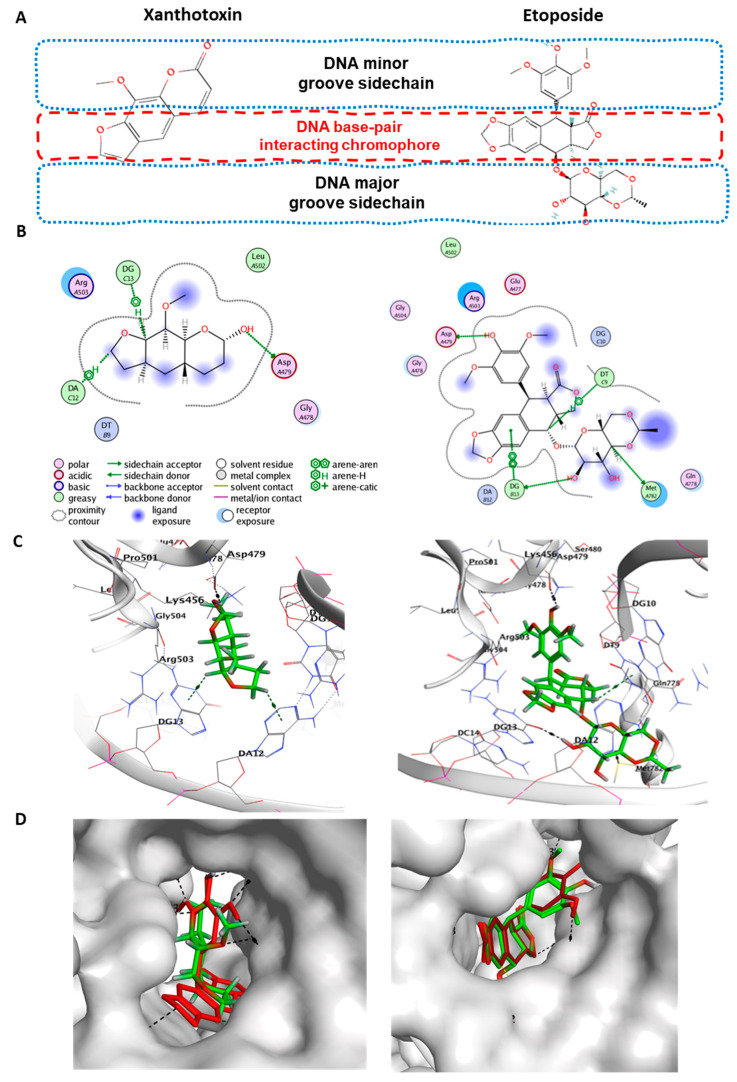
Binding modes of xanthotoxin and etoposide after docking in the active site of topoisomerase II (PDB code: 3QX3). (**A**) The chemical structure of docked compounds xanthotoxin and etoposide. (**B**) Two-dimensional (2D) structures of ligand interactions. (**C**) 3D binding mode illustrating their interactions both with the same amino acid Asp479 inside the human Topo II–DNA complex active site (rendered in stick mode and with hydrogen bonds represented in black dashed lines) and H–pi interactions with DNA nucleotides (in blue dashed lines). (**D**) 3D positioning and 3D molecular surface diagrams of xanthotoxin and docked etoposide (green sticks) along with the native co-crystalized ligand etoposide (red sticks) inside its active site.

To elucidate the mechanism of induction of apoptosis that was induced by xanthotoxin, we investigated the effects of xanthotoxin on targeting the enzyme topoisomerase IIb, which is vital for DNA replication, chromosome condensation, and chromosome segregation, and its inhibition leads to the induction of apoptosis in proliferating cancer cells [23]. Therefore, a molecular docking study was performed to investigate the plausible binding interaction of xanthotoxin with the key amino acid in the active site of topoisomerase II and compared with that of its co-crystalized inhibitor etoposide (PDB code: 3QX3). The bound etoposide interacts extensively with both the protein and DNA through the drug’s polycyclic aglycone heterocyclic rings sitting between DNA base pairs and the glycosidic group and the front ring protruding toward the DNA major and minor grooves, respectively. The key drug-contacting residue for interacting DNA–Topo II complex with the bound etoposide consists of amino acid residues of Asp479, Arg503, Gln778, Leu502, and Met782 as well as the nucleotides of Cyt8, Thy9, Cyt11, Gua13, and Ade12 [24]. Our results indicated that xanthotoxin was docked with the crystal structure of topoisomerase IIb with an energy score of −5.72 kcal/mol, which was slightly higher compared to that of etoposide (−7.31 Kcal/mol) (Table 2). The molecular interaction of xanthotoxin revealed strong interactions with the key amino acid Asp479 that the co-crystalized inhibitor (etoposide) bonded with hydrogen-bond inside the active site of human Topo II–DNA complex (Figure 7). In addition, the presence of two H–π interactions of xanthotoxin with DG13 and DA12 confirmed the stable pose of the xanthotoxin in the binding pocket of topoisomerase IIb.

## 3. Discussion

Various approaches aimed to discover new drug candidates either with cytotoxic activity or by enhancing the selectivity and activity of the present anticancer drugs [4,25]. Therefore, our study aimed to identify compounds with anticancer activity from natural plant sources wildly growing in Egypt. *A. majus*, an indigenous plant in the Delta regions of Egypt, has been used traditionally for hundreds of years as a treatment of various diseases [10]. Recently, *Ammi visnaga* extract was studied, and it was reported that it exhibited anticancer activity against hepatic cancer [26]. These data motivated us to investigate the anticancer activity of *A. majus* and to use bio-guided fractionation methods to further specify the active compound(s) in its extract implicated in this activity against liver cancer cells. The sulforhodamine B (SRB) assay was applied to determine the antiproliferative activity of the different isolated compounds, and it was found that the coumarin xanthotoxin has the highest cytotoxic activity, of almost three-fold and one-fold greater compared to total AME and CH_2_Cl_2_ fractions, respectively, approaching that of doxorubicin with IC_50_ 6.9 μg/mL. Xanthotoxin is a major coumarin found in *A. majus* fruits that is currently used in the treatment of skin diseases such as vitiligo and psoriasis and some cutaneous lymphoma [27,28]. It was previously reported to have cytotoxic activity on various cell lines [29,30]. However, to the best of our knowledge, this is the first report on the antitumor activity of xanthotoxin isolated from *A. majus* fruits against liver cancer cells. Interestingly, AME extract showed low cytotoxicity against normal human fibroblastic (NHF1) cell line, implying a good cytocompatibility with normal human cells.

Hepatocellular carcinoma develops in the situation of chronic liver disease upon complex interactions between the environmental factors and the host that causes genetic alterations, leading to unlimited cell proliferation, dysregulated apoptosis, promoted autophagy, and enhanced tissue invasion and metastasis [31]. Therefore, the underling mechanism(s) of xanthotoxin on the liver cancer cell cycle kinetics, programmed cell death (apoptosis), and cellular autophagy besides elucidation of the mechanism of induction of apoptosis using molecular docking on topoisomerase IIb enzyme were investigated herein. Apoptosis is defined as the programmed cell death of a cell in any pathological condition when mediated by internal or external stimuli [32]. On the other hand, autophagy can play either a pro-survival or pro-death role in malignant neoplasm cells [33]. Therefore, deciphering cell death signaling pathways could contribute in the development of new targeted therapies [32]. The present study showed that xanthotoxin exerted different molecular mechanisms by altering the cell cycle profile as it stopped the cell proliferation of HepG2 cells via cell cycle arrest at the G2/M phase and by induction of apoptosis. Xanthotoxin also induced apoptosis, as we found significantly higher percentages of apoptotic cells using annexin-V apoptosis assay in HepG2 cells treated with xanthotoxin.

A mounting body of evidence indicates that cancer cells use autophagy as a cytoprotective mechanism by limiting tumor necrosis and inflammation through blocking the signaling pathways of intrinsic and extrinsic apoptosis upon autophagy activation [33], which results in adaptation and survival of tumor cells under hypoxic and inflammatory microenvironment such as in case of established HCC [34]. Therefore, autophagy is considered a potential therapeutic target in adjuvant chemotherapy [35]. Our data revealed that xanthotoxin inhibited autophagic flux in HepG2 cells, which may has caused activation in the apoptosis pathway, the matter that may account for the significantly higher percentages of apoptotic HepG2 cells after treatment with xanthotoxin.

Our results indicate that xanthotoxin is exhibiting anticancer effects with good biocompatibility toward normal human cells. This variation in response to xanthotoxin between normal and cancer cells could be related to one of the main characteristics of cancer cells over normal ones, that is, their high expression of the relaxing enzyme topoisomerase II [36]. Topoisomerase II is an essential DNA helix nuclear enzyme that segregates newly replicated chromosome pairs and causes relaxation of DNA superhelicity by untangling intertwined DNA strands before cell division through transiently breaking and re-ligating DNA strands. Chemotherapeutic agents that inhibit topoisomerase II such as etoposide acts by stabilizing a normally transient DNA–topoisomerase II complex, leading to increased double-stranded DNA breaks, which triggers as a result the activation of cell death pathways [37,38]. Interestingly, the molecular docking analysis revealed that xanthotoxin was docked with the crystal structure of topoisomerase II with an energy score of −5.72 kcal/mol, which was slightly higher compared to that of etoposide (−7.31 Kcal/mol). However, we found that xanthotoxin interacts with the key amino acid Asp479 in a similar fashion to that of etoposide. These findings imply that xanthotoxin has great potential to inhibit topoisomerase IIb. Since topoisomerase IIb enzyme is implicated in DNA recombination, replication, transcription, and repair processes, compounds that inhibit it such as xanthotoxin are considered broad-spectrum anticancer agents.

## 4. Materials and Methods

### 4.1. Chemical Studies

#### 4.1.1. Plant Material

Fruits of *A. majus* were obtained in November 2017 near (30.3799° N, 31.4544° E) from Arab Company of Pharmaceuticals and Medicinal Plants (Mepaco-Medifood) El-Sharkya, Egypt. They were kept at room temperature in well-sealed containers. Dr. Mokhtar Bishr, Mepaco Company’s Technical Director, identified plant samples and kept them in Mepaco Company (El-Sharkya, Egypt) herbarium with the number RD-235-018.

#### 4.1.2. Extraction and Fractionation of the Plant Material

The air-dried *A. majus* fruits (1.5 kg) were ground into coarse powder and successively macerated with 70% methanol at room temperature until exhaustion. The methanol extract was concentrated by evaporation at 50 °C under reduced pressure, yielding 250 g of sticky dark brown dry extract (AME), which was kept in an amber, glass, well-closed container in the refrigerator until use. The total extract (200 g) was fractionated using n-hexane (3 × 500 mL), followed by CH_2_Cl_2_ (3 × 500 mL) and finally EtOAc (3 × 500 mL). The pooled fractions were separately evaporated to dryness under vacuum to yield dry weights of hexane fraction (38.54 g), CH_2_Cl_2_ fraction (50 g), EtOAc fraction (7.36 g), and methanol (25.21 g).

#### 4.1.3. Purification and Isolation of the Compounds

The dichloromethane residue (50 g) was chosen for further investigation based on the biological activity and the published data regarding the presence of coumarins [12,39]. Vacuum liquid chromatography (VLC) was used for the fractionation, and the column was packed with silica gel 60H. A gradient elution system was carried out using n-hexane/CH_2_Cl_2_ and CH_2_Cl_2_/methanol systems.

Vacuum liquid chromatography eluates (50 mL) were investigated using TLC, silica gel 60F 254, and precoated plates by different solvent systems with different polarities. Two fractions with the clearest and major spots named fraction A and fraction B were chosen for further purification using Puriflash 4100 system (Interchim; Montluçon, France), consisting of 25 g-flash-NP column (30 µm), a mixing HPLC quaternary pump, a PDA–UV-Vis detector 190–840 nm, a fraction collector, and a sample loading module. For system controlling and process monitoring, Interchim Software 5.0 was used. Elution was done using normal phase gradient elution systems, and the samples were collected in test tubes, and then the solvents were evaporated.

The isolated compounds were subjected to identification and structure elucidation using NMR spectra recorded on a Bruker AVANCE HD III 400 MHz spectrometer (Bruker, Fällanden, Switzerland).

### 4.2. Biological Evaluation

#### 4.2.1. Cell Culture

Normal human fibroblast (NHF1) and human liver cancer cell line (HepG2) obtained from the American Type Culture Collection (ATCC, Manassas, VA, USA) were used for investigating the cytocompatibility and the cytotoxicity of the tested compounds. Cells were maintained in complete DMEM culture medium, supplemented with 10% heat-inactivated fetal bovine serum, and 1% penicillin/streptomycin. Cells were kept passaging in subconfluence phase in humidified 5% CO_2_ (*v*/*v*) atmosphere at 37 °C.

#### 4.2.2. Cytocompatibility and Cytotoxicity Assessments

The tested compounds were investigated for their cytocompatibility and cytotoxicity against Normal Human Fibroblast (NHF1) and liver cancer (HepG2) cell lines, respectively, using SRB assay, as previously described [40]. Briefly, exponentially growing cells were trypsinzed by 0.25% Trypsin-EDTA and seeded in 96-well plates at 1000–2000 cells/well. Cells were treated thrice with serial concentrations of the isolated compounds for 48 h and subsequently fixed with TCA (10%) for 1 h at 4 °C. After washings with water several times, cells were stained with 0.4% SRB solution for 10 min. at room temperature in a dark place and subsequently washed with 1% glacial acetic acid. Plates were left overnight for drying, then Tris-HCl was added to dissolve the SRB within stained cells. The intensity of the developed color was measured at 540 nm with a microplate reader (Spectramax^®^ M3, Molecular devices, San Jose, CA, USA). The percentage of cell survival was calculated as follows: Survival fraction = OD. (Treated cells)/OD (control cells). Cells cultured with DMSO plus medium alone were counted as negative control, and those with doxorubicin were used as a positive control. Results were depicted as % viability relative to 100% of controls. IC_50_ is defined as the compound concentration required to reduce absorbance by 50%. The experiments were repeated three times, and data were represented as mean ± SD of three replicates.

#### 4.2.3. Cell Cycle Kinetics Analysis

The effect on cell cycle distribution of the most efficient antitumor compound was tested, as previously described [41]. Briefly, cells were incubated with IC_50_ of the test compounds for 48 h. Treated cells were detached by trypsinization, washed twice with ice-cold PBS and resuspended in 0.5 mL PBS. Cells were fixed by adding 2 mL of 70% ice-cold drop wisely while mixing, and cells were left in ethanol solution for an hour at 4 °C. Cells were then washed and stained with 1 mL of staining buffer containing 50 μg/mL RNAase A and 10 μg/mL propidium iodide (PI). Cells were incubated in the dark at room temperature for 20 min, then they were analyzed for cell cycle kinetics using flow cytometry (ACEA Novocyte™, ACEA Biosciences Inc., San Diego, CA, USA). For each sample, 10,000 events were acquired and analyzed for PI fluorescent signals using FL2 detector (λex/em 535/617 nm). The percent of cells in each cell cycle phase was calculated using ACEA NovoExpress™ software (ACEA Biosciences Inc., San Diego, CA, USA). Each treatment was repeated three times, and data were presented as mean ± SD of the three replicates.

#### 4.2.4. Apoptosis Analysis by Flow Cytometry

The effects of the most efficient antitumor compound on the induction of apoptosis and necrosis were determined as described previously [42]. Briefly, cells were treated with IC_50_ of tested compounds for 48 h. After treatment, cells were detached by trypsinization, washed twice with ice-cold PBS, and resuspended in 0.5 mL of annexin/V-FITC/PI solution for 30 min. in the dark according to the manufacturer protocol (Abcam Inc., Cambridge Science Park, Cambridge, UK). Stained cells were analyzed for FITC and PI fluorescent signals using FL1 and FL2 signal detector, respectively (λex/em 488/530 nm for FITC and λex/em 535/617 nm for PI), using ACEA Novocyte™ flow cytometer (ACEA Biosciences Inc., San Diego, CA, USA). For each sample, 10,000 events were acquired, and positive FITC and/or PI cells were quantified by quadrant analysis and calculated using ACEA NovoExpress™ software (ACEA Biosciences Inc., San Diego, CA, USA). Each treatment was repeated three times, and data were presented as the mean (±SD) of the three replicates.

#### 4.2.5. Autophagy Assay

Autophagic cell death is quantitatively assessed using acridine orange lysosomal stain coupled with flow cytometric analysis [43]. After treatment with test compounds for 48 h and chloroquine (10 µM) for 48 h as positive control, cells (10^5^ cells) were collected by trypsinization and washed twice with ice-cold PBS (pH 7.4). Cells were stained with acridine orange (10 µM) and incubated in the dark at 37 °C for 30 min. Stained cells were analyzed for acridine orange fluorescent signal efflux using flow cytometer (ACEA Novocyte™, ACEA Biosciences Inc., San Diego, CA, USA) with FL1 signal detector (λex/em 488/530 nm). For each sample, 10,000 events were acquired, and mean fluorescent intensities (MFI) were quantified using ACEA NovoExpress™ software (ACEA Biosciences Inc., San Diego, CA, USA).

### 4.3. Molecular Docking Analysis

To investigate the possible mechanism of the anticancer effect of tested compounds, their ability to target topoisomerase II (an enzyme essential for DNA replication) was tested by a molecular docking study using Molecular Operating Environment (MOE^®^) (MOE version 2014.09, Chemical Computing Group Inc., Montreal, QC, Canada), as previously described, with minor modifications [37]. Briefly, the target compounds were constructed into 3D models using the builder interface of the MOE program and then subjected to a conformational search. All conformers were subjected to energy minimization and partial charges calculations. The obtained database was then saved as an MDB file to be used in the docking calculations. The X-ray crystallographic structures of the human DNA topoisomerase II-beta (in complex with DNA and etoposide) enzyme was obtained from RCSB Protein Data Bank [PDB code: 3QX3]. The enzyme was prepared by adding hydrogen atoms to the system with their standard geometry. MOE Alpha Site Finder was used for the active site search in the enzyme structure using all default items. Dummy atoms were created from the obtained alpha spheres. The MDB files of ligands to be docked were loaded, and docking calculations were performed using the default docking parameters in the MOE-Dock suite, as previously reported [44]. The obtained poses were studied, and the poses showing best ligand–enzyme interactions were selected and stored for energy calculations using the triangle matcher placement method and the London dG scoring system, which calculates the binding free energy of the ligand for each pose.

### 4.4. Statistical Analysis

Data are presented as mean ± SD. Analysis of variance (ANOVA) followed by Tukey’s post hoc test was used for testing the significance using SPSS software for Windows, version 20.0. *p*-value of 0.05 or less was considered significant.

## 5. Conclusions

*A. majus*, a wild endemic plant in Egypt with wide pharmaceutical potential and medicinal features producing effective active constituents such as coumarins, has exhibited versatile usage in medicine. In this study, the most active compound in AME was xanthotoxin. Xanthotoxin is a furanocoumarin derivative and proved to have cytotoxic activity against HepG2. Moreover, xanthotoxin demonstrated topoisomerase II inhibitory activity, which may be the underlying mechanism behind its potential anticancer activity. More studies are needed to enhance the knowledge of this compound for future developments of novel medicine not only in cancer treatment but also in other diseases such as cardiovascular, inflammatory, and neurological diseases and to evaluate the effect of its combinations with other currently approved drugs to overcome cancer cells’ resistance to treatments. Furthermore, research is needed to find simple, natural, more cost-effective, and beneficial anticancer pharmaceutical products.

## Figures and Tables

**Table 1:** (**a**) ^1^H-NMR and ^13^C-NMR/APT-NMR of coumarin compounds isolated from *A. majus*. fruits dichloromethane fraction (**b**) ^1^H-NMR APT-NMR of β-sitosterol isolated from *A. majus* fruits dichloromethane fraction.

**(a)**								
**Position**	**C1**		**C3**	**C4**		**C5**	**C6**	
	**δ_H_**	**δ_C_**	**δ_H_**	**δ_H_**	**δ_C_**	**δ_H_**	**δ_H_**	**δ_C_**
1	-	-	-	-	-	-	-	-
2	-	161.84	-	-	169.90	-	-	163.
3	6.38 (d, 12)	113.82	6.08 (d, 7.2)	6.40 (d, 8.5)	100.12	6.40 (d, 9.5)	7.05 (d, 7.2)	105.50
4	7.78 (d, 12)	142.50	7.62 (d, 8.5)	7.80 (d, 7.9)	144.83	7.79 (d, 9.6)	7.61 (d, 7.2)	145.21
5	7.37 (s)	114.82	7.05 (d, 8.0)	6.85 (s)	130.55	-	7.28 (s)	130.21
6	-	126.26	7.27 (d, 9.3)	-	127.94	-	-	124.82
7	-	147.16	-	-	164.55	-	-	163
8	-	132.80	-	7.72 (s)	111.50	7.38 (s)	7.28 (s)	117
1′	-	-	3.57 (d, 7.7)	-	-	-	-	-
2′	7.70 (d, 4)	147.80	5.37 (m)	4.21 (m)	95.90	7.72 (d, 2.3)	4.76 (m)	95
3′	6.83 (d, 4)	107.32	-	2.42 (d, 0.8)	21.50	6.84 (d, 2.2)	2.35 (d, 0.7)	41
4′	4.30 (s)	50.10	1.70 (s)	-	62.10	-	-	130.15
5′	-	-	4.20 (s)	1.28 (s)	19.97	-	6.07 (s)	115.47
6′	-	-	-	1.65 (s)	19.97	-	1.27 (s)	20.10
1a	-	145.26	-	-	145.51	-	-	110.34
4a	-	116.18	-	-	107.50	-	-	142.96
1″	-	-	-	-	-	1.57 (s)	-	-
2″	-	-	-	-	-	5.39 (m)	-	-
3″	-	-	-	-	-	-	-	-
4″	-	-	-	-	-	1.28 (s)	-	-
5″	-	-	-	-	-	4.33 (d, 2.3)	-	-
**(b)**								
**Position**					**C2**			
					**δH**			
**1**					1.51 (m)			
**2**					1.60 (m)			
**3**					3.55 (m).			
**4**					2.01 (d, 5.1).			
**5, 10, 13**					No proton			
**6**					5.37 (m)			
**18**					0.83 (s)			
**19**					1.03 (s)			
**21**					0.94 (d, 6.5)			
**26**					0.88 (d, 8.0)			
**27**					0.85 (d, 1.8)			
**28**					1.43–1.24 (m)			
**29**					1.24 (m)			

**Table 2:** The binding scores and modes of *Xanthotoxin* and the docked co-crystallized inhibitor etoposide inside the binding site of the topoisomerase IIb.

Compound	^a^ S	^b^ RMSD (Å )	Amino Acid/Bond	Distance (Å)	E (Kcal/mol)
Xanthotoxin	−5.72	1.51	Asp479/H–donor	2.87	−3.01
			DA12/H–pi	3.98	−1.20
			DG13/H–pi	3.72	−0.07
Etoposide	−7.31	1.93	ASP479/H–donor	2.56	−3.0
			MET782/H–donor	3.26	−0.4
			DG13/H–donor	2.96	−2.8
			DG13/pi–pi	2.72	−3.1
			DA12/H–pi	3.79	−1.0

^a^ S: the score of a ligand inside the binding pocket of the protein (Kcal/mol), ^b^ RMSD: the root mean square deviation of distance between two crystal structures in angstrom (Å).

## Data Availability

Data is contained within the article and supplementary materials.

## Supplementary Materials

The following supporting information can be downloaded online. Spectral data analysis (^1^H- and ^13^C-NMR) of the isolated compounds is available in a supplementary file.

## Sample Availability

Samples of the compounds are not available from the authors.
