# Supplementary material for: Potential Anticancer Activity of the Furanocoumarin Derivative Xanthotoxin Isolated from Ammi majus L. Fruits: In Vitro and In Silico Studies
Source: Molecules. 2022 Jan 29;27(3):943. doi: 10.3390/molecules27030943 (PMC8839012; doi:10.3390/molecules27030943)
Supplement: Supplementary file 1 [file molecules-27-00943-s001.zip › molecules-1556900-supplementary.pdf]

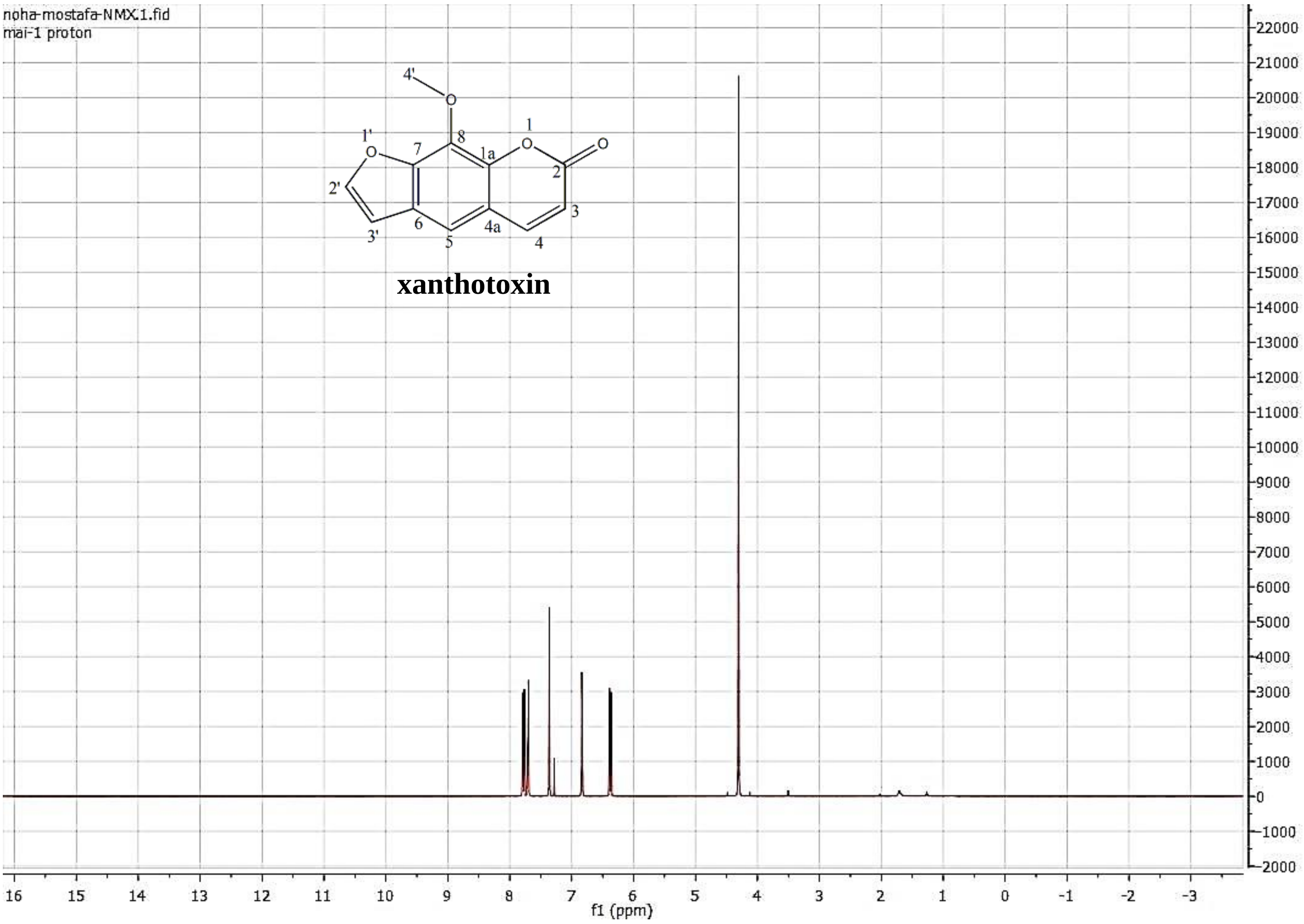

^{1}H-NMR spectrum of compound C-1 ($CDCl_3$, 400 MHz)

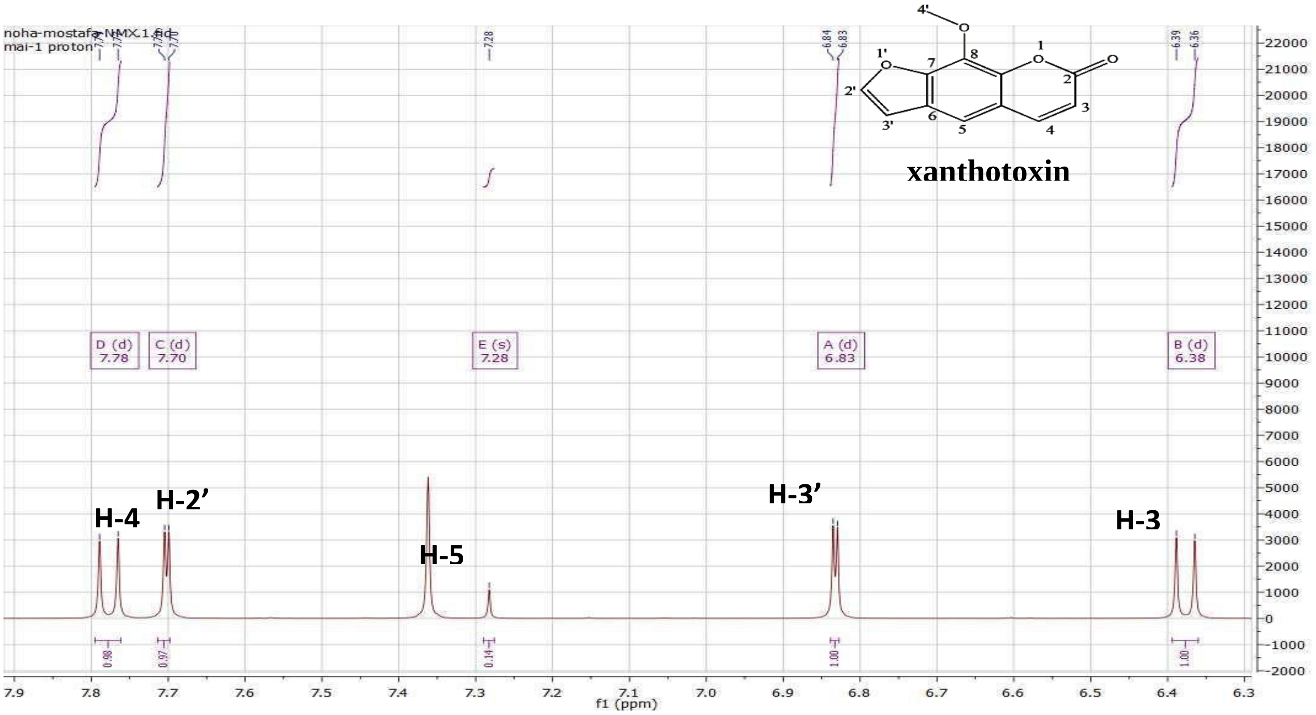

^{1}H-NMR magnification spectrum of compound C-1 ($CDCl_3$, 400 MHz)

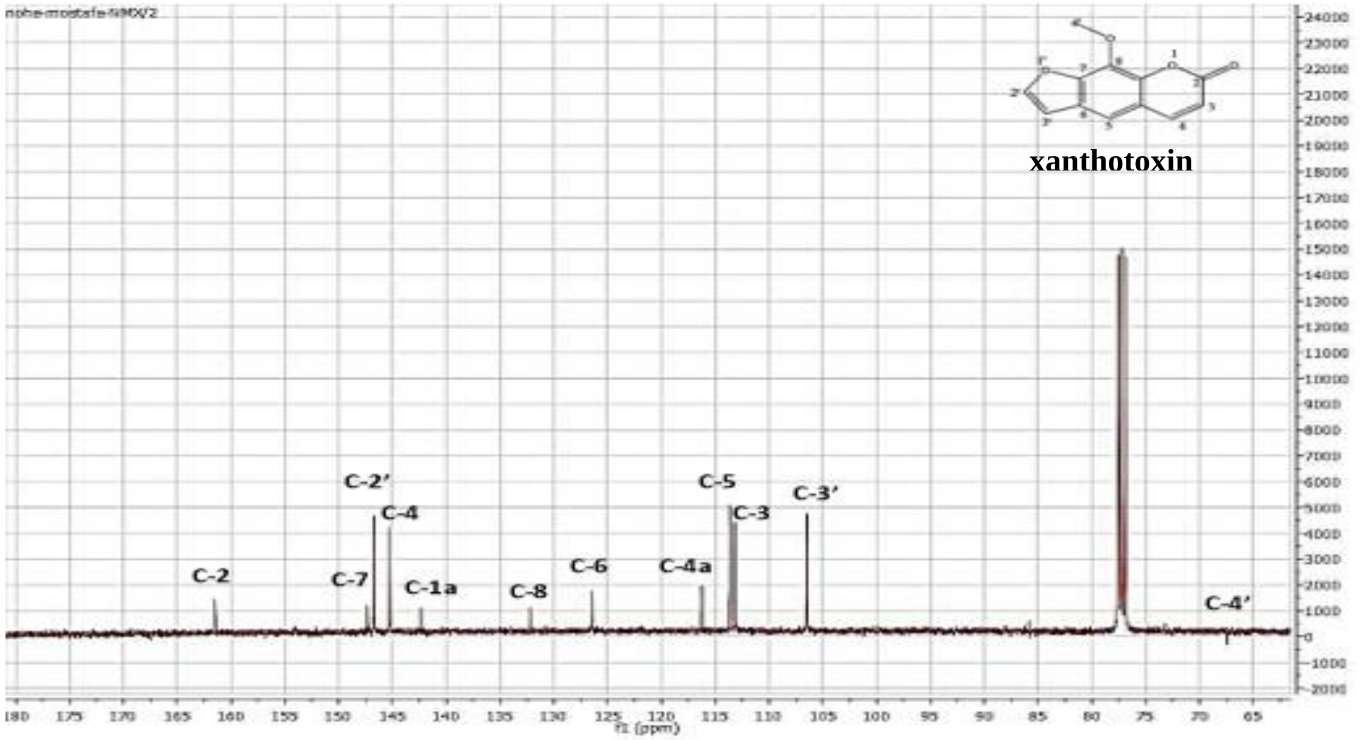

^{13}C-NMR magnification spectrum of compound C-1 ($CDCl_3$, 400 MHz)

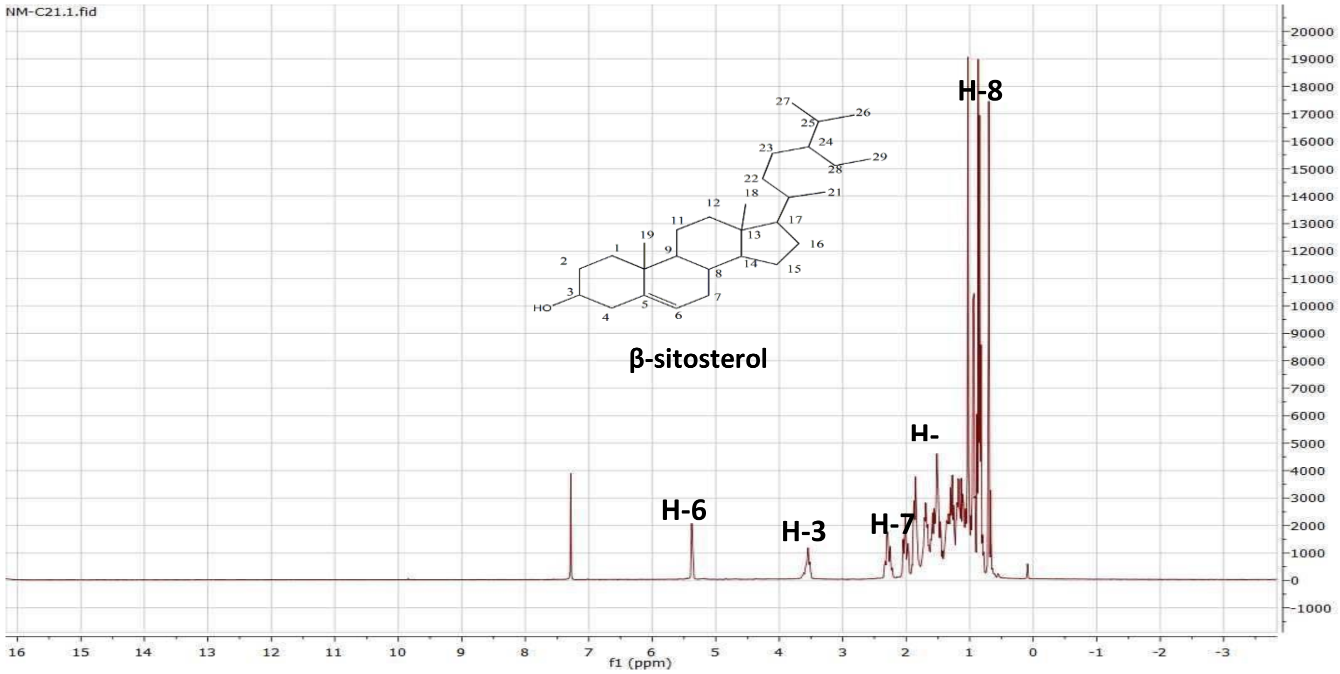

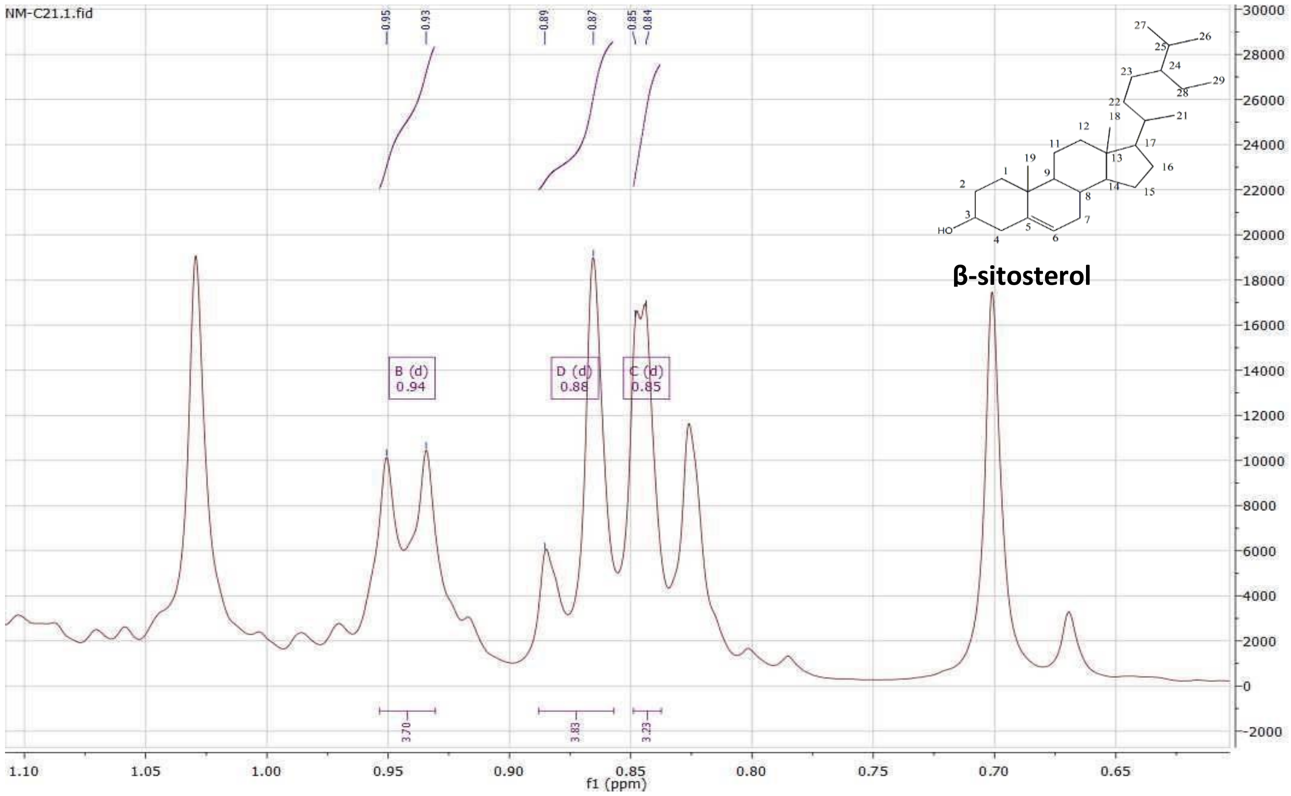

^{1}H-NMR spectrum of compound C-2 ($CDCl_3$, 400 MHz)

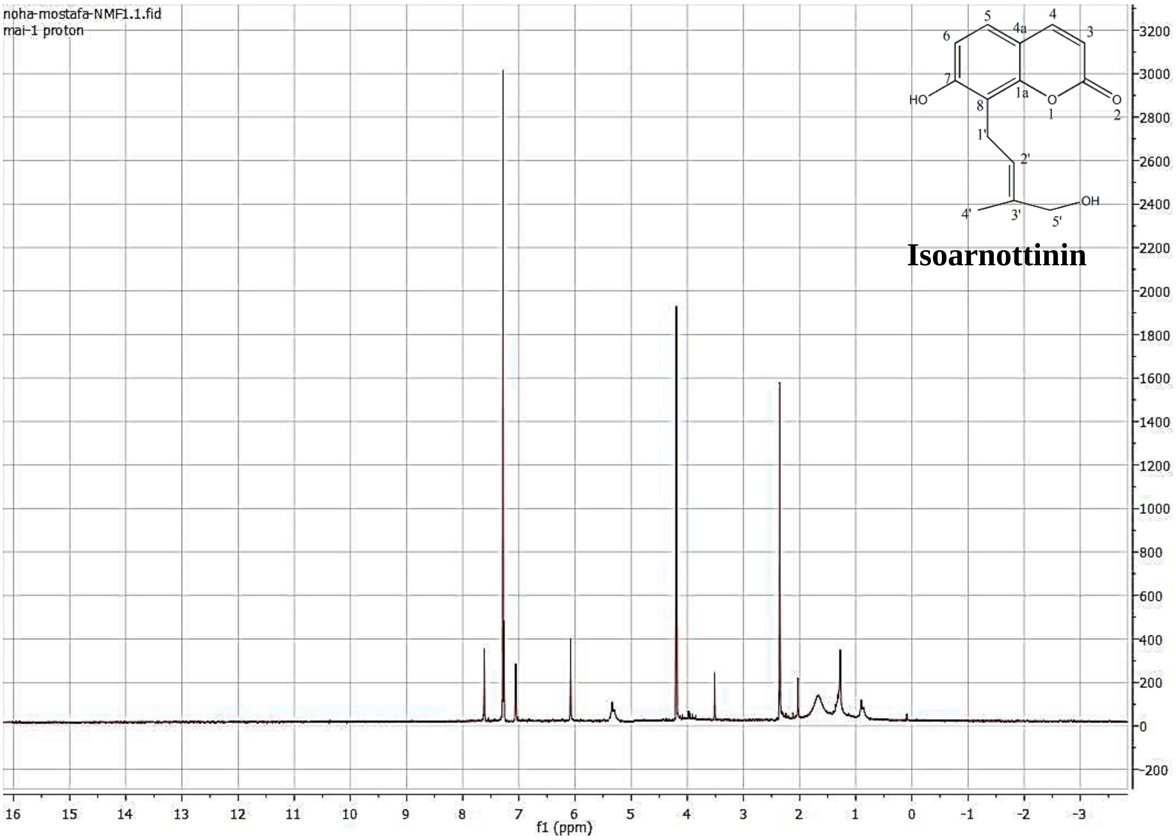

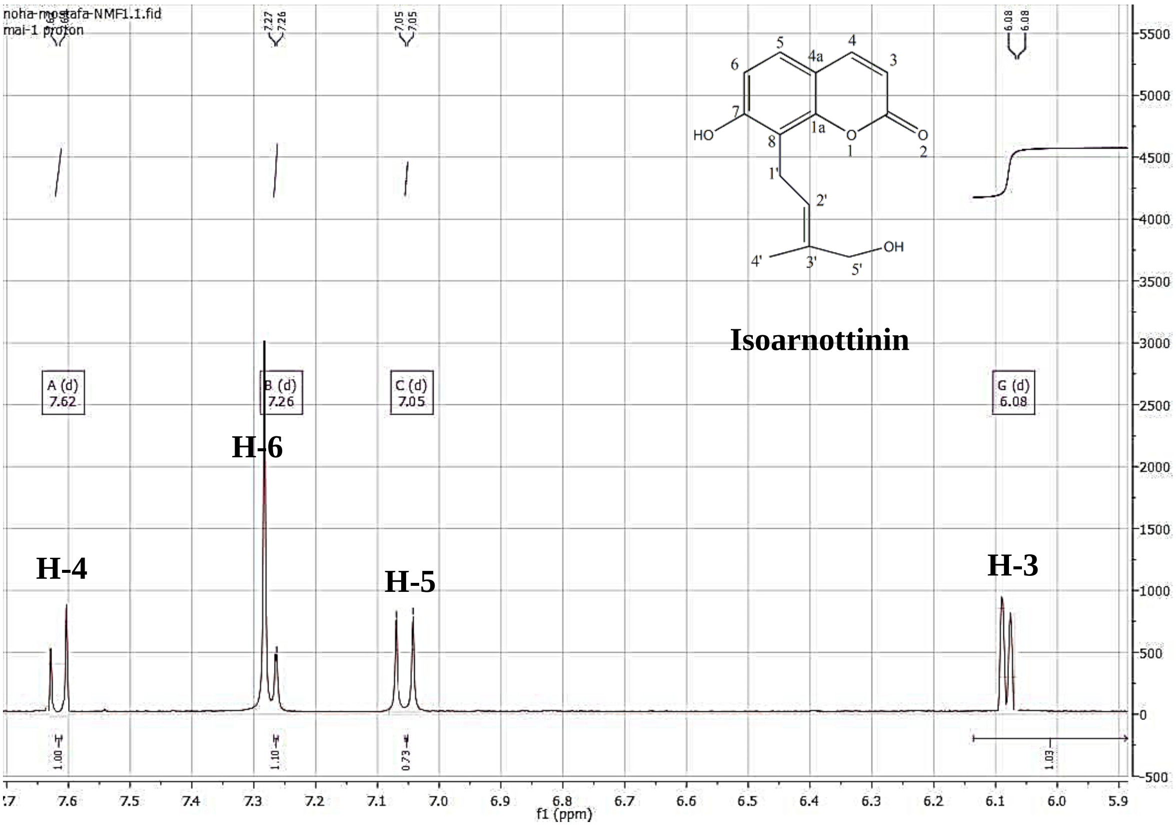

^{1}H-NMR spectrum of compound C-3 ($CDCl_3$, 400 MHz)

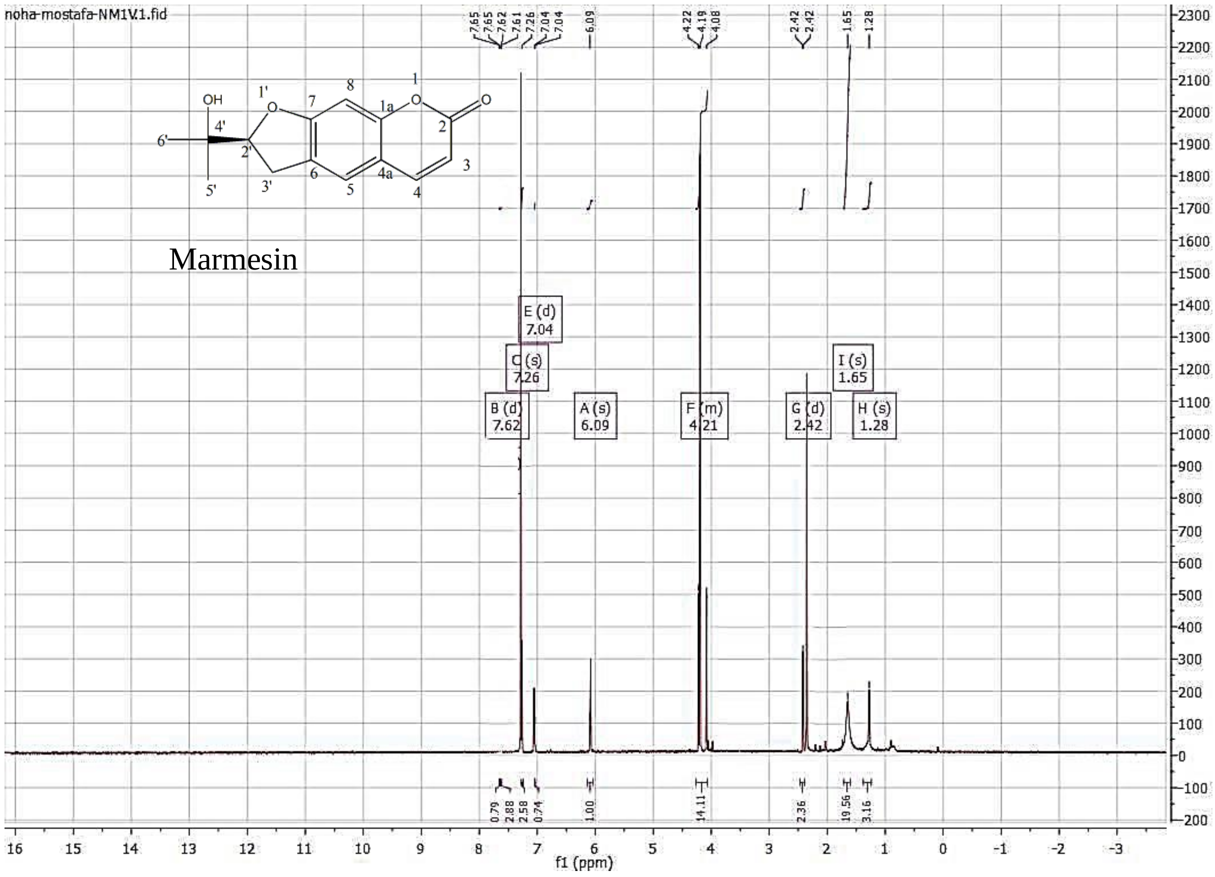

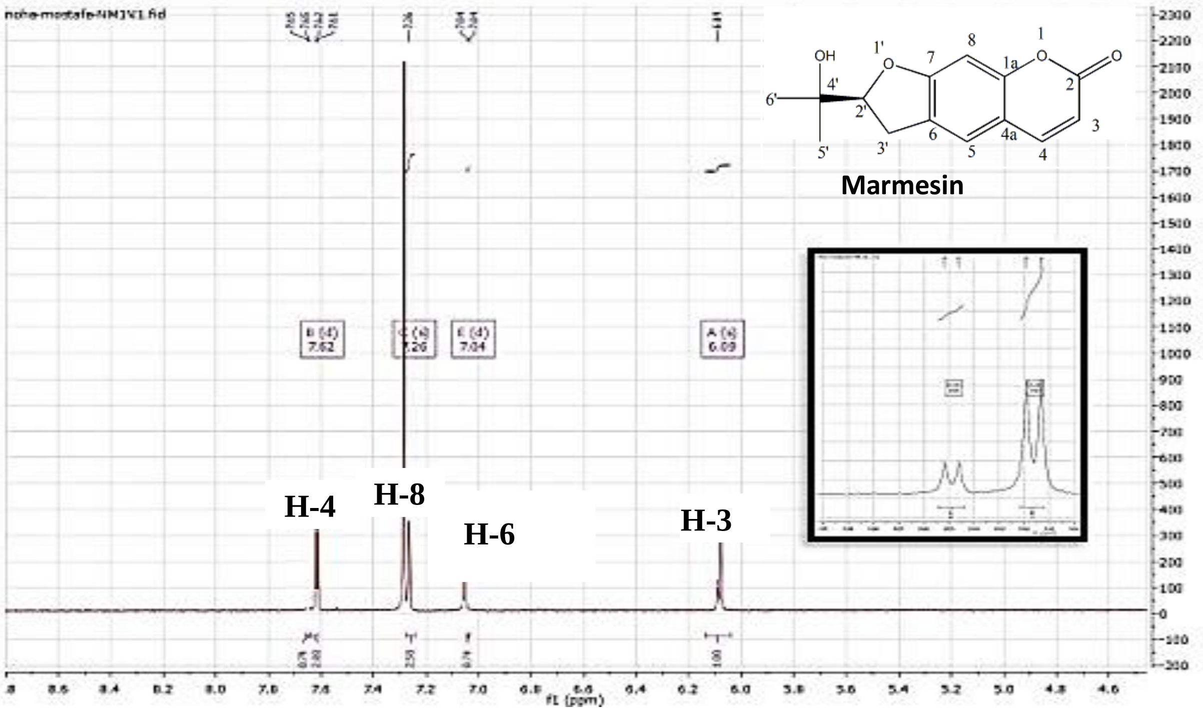

^{1}H-NMR spectrum magnification of compound C-4, ($CDCl_3$, 400 MHz)

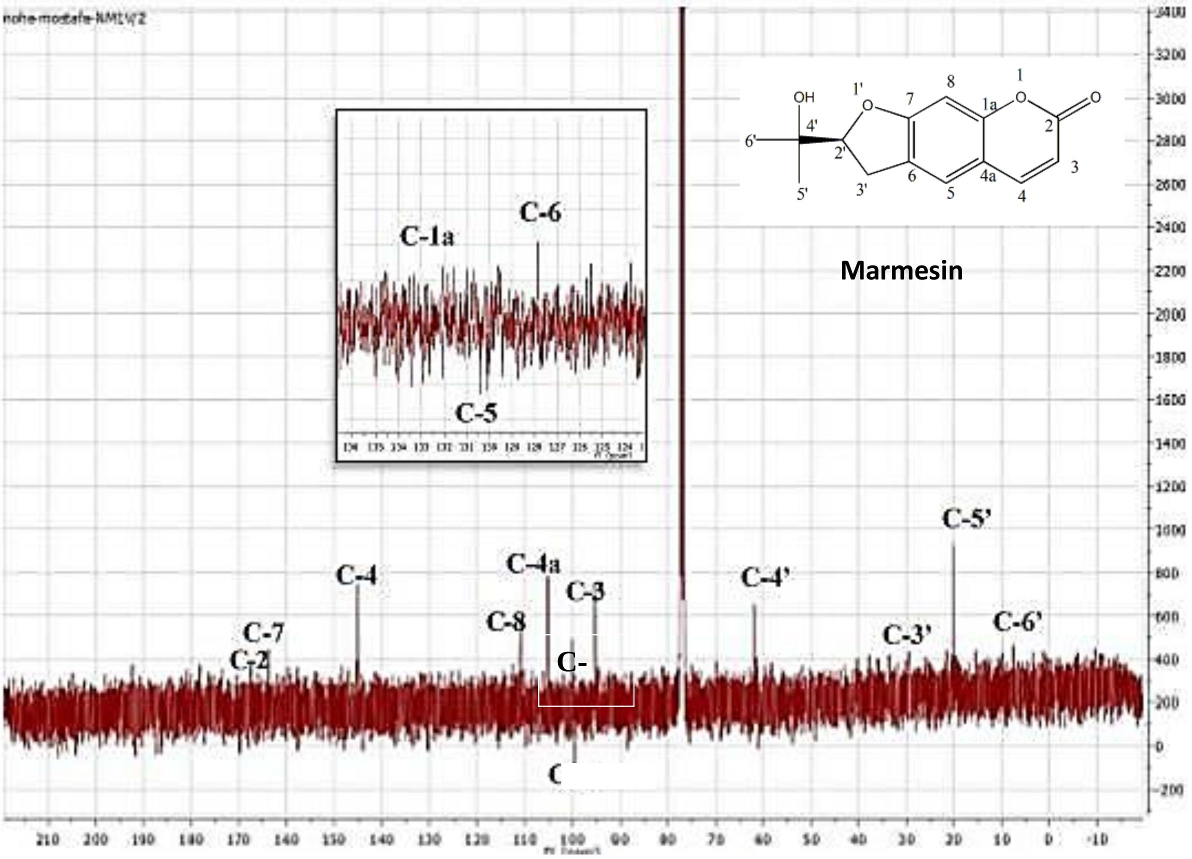

^{13}C-NMR spectrum of compound C-4, ($CDCl_3$, 400 MHz)

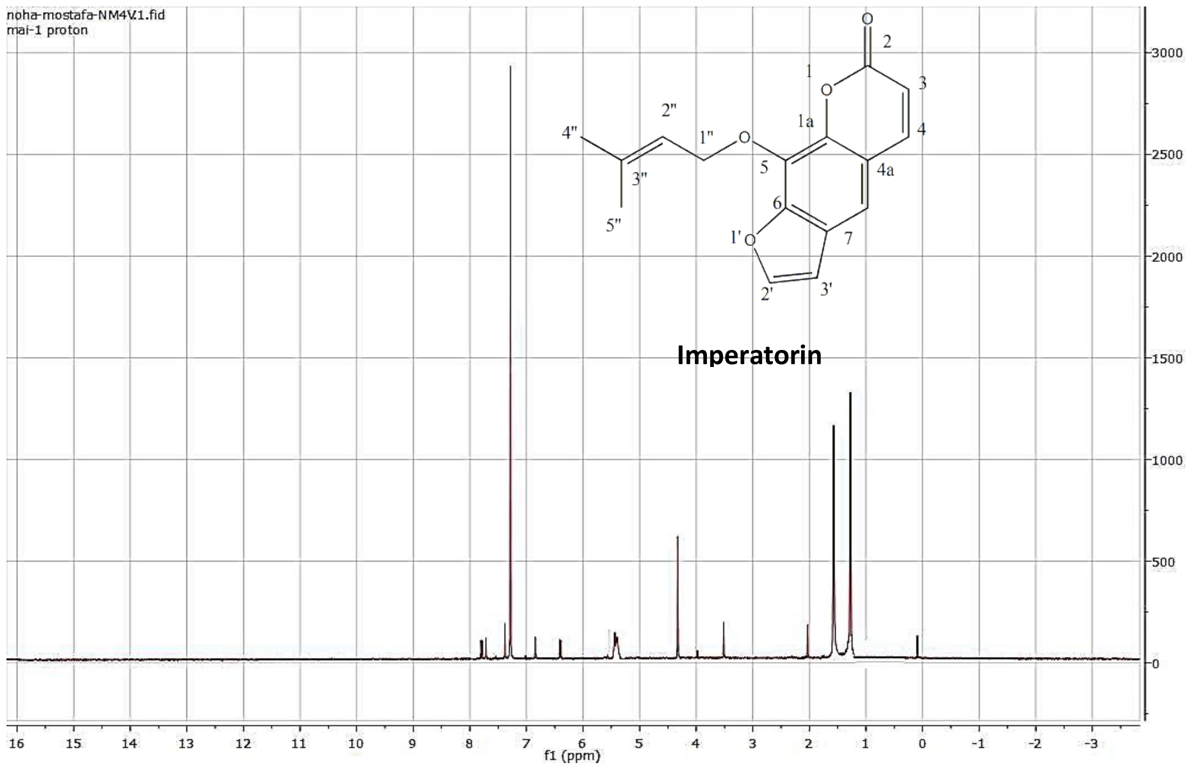

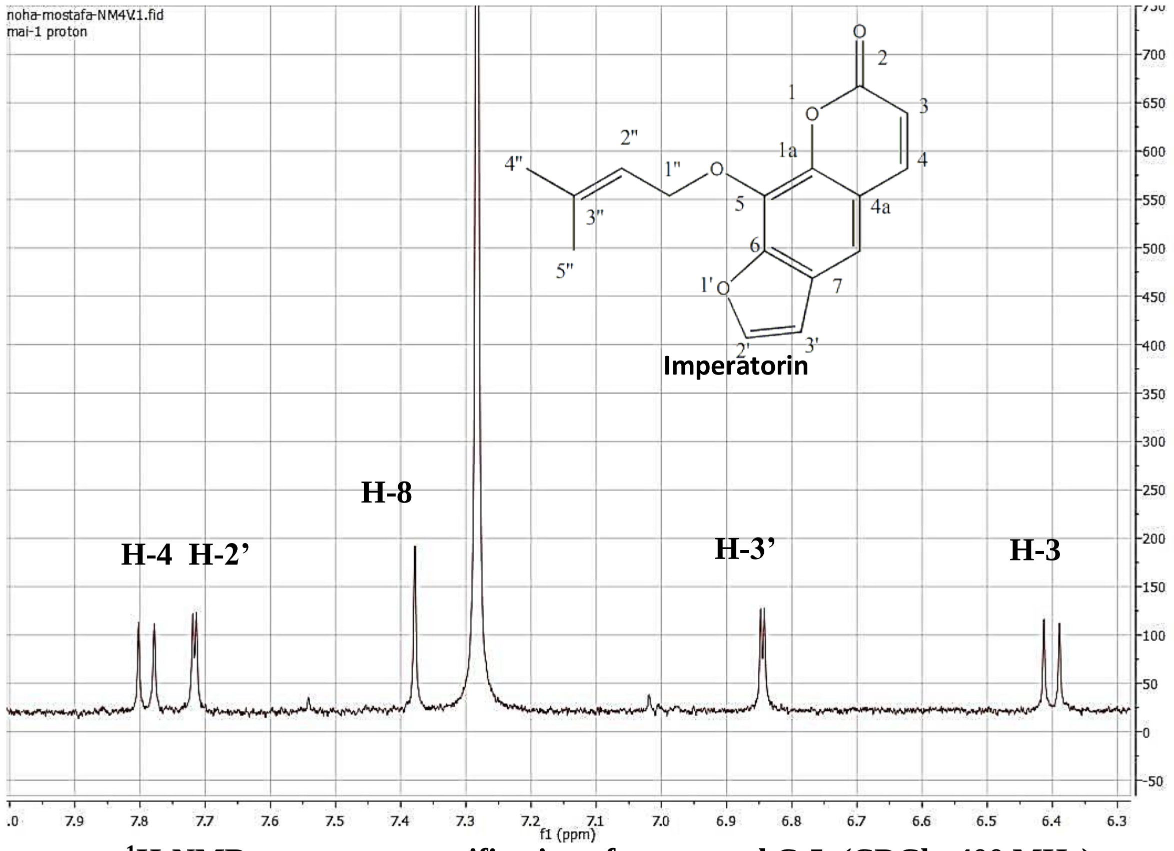

^{1}H-NMR spectrum magnification of compound C-5, ($CDCl_3$, 400 MHz)

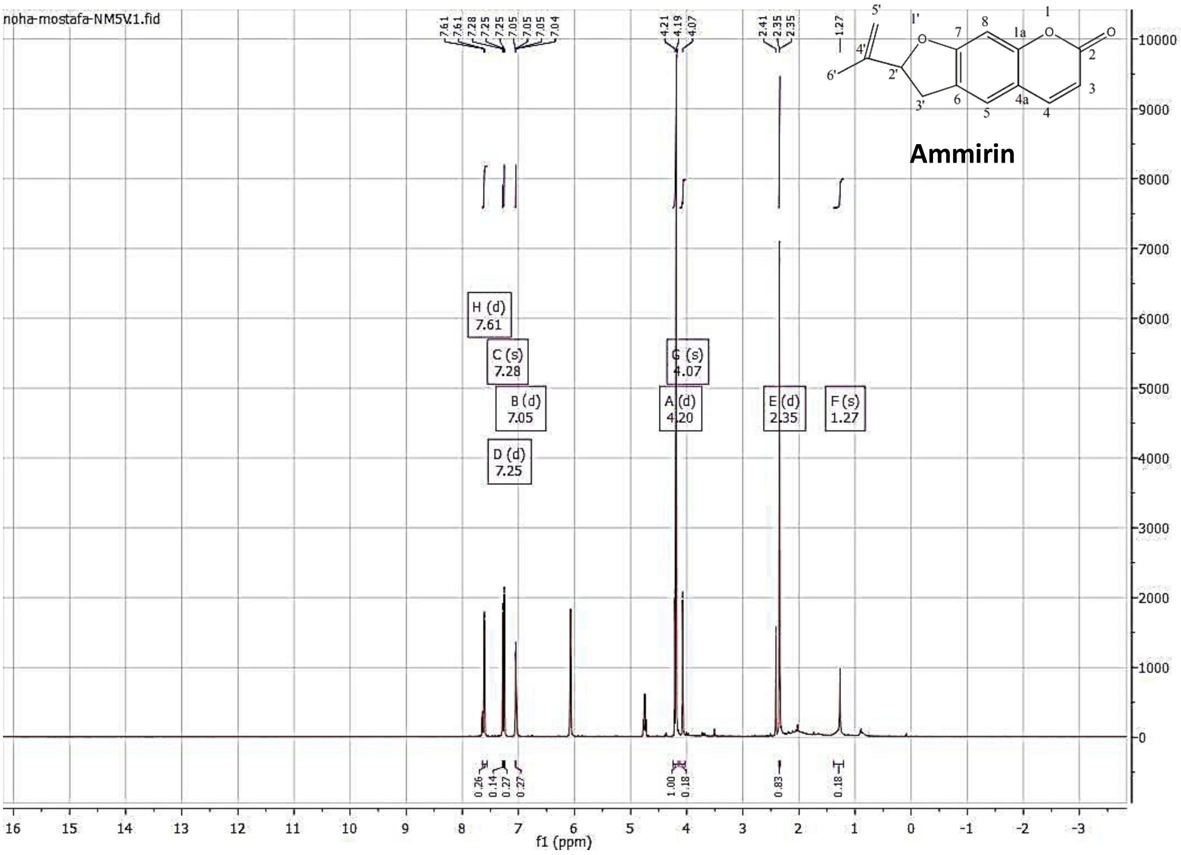

^{1}H-NMR spectrum of compound C-6 CDCl3, 400 MHz

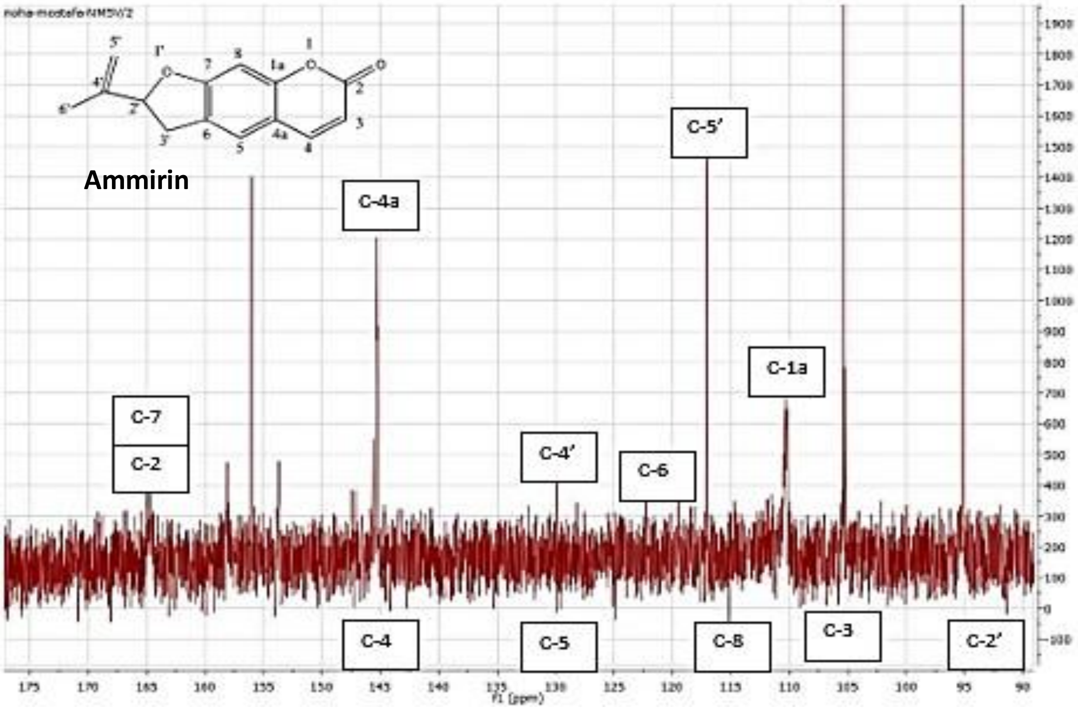

^{13}C-NMR spectrum magnification of compound C-6 CDCl3, 400 MHz
